# Calculating an institutional personal protective equipment (PPE) burn rate to project future usage patterns during the 2020 COVID-19 pandemic

**DOI:** 10.1017/ice.2020.190

**Published:** 2020-05-04

**Authors:** Sumanth Raja, Harsh H. Patolia, Anthony W. Baffoe-Bonnie

**Affiliations:** 1 Carilion Clinic Roanoke Memorial Hospital, Roanoke, Virginia; 2 Virginia Tech Carilion School of Medicine, Roanoke, Virginia; 3 Carilion Clinic Infection Control Department, Roanoke, Virginia

*To the Editor—*The COVID-19 pandemic of 2020, caused by a severe coronavirus strain named SARS-CoV-2, and has created shortages of personal protective equipment (PPE).^1^ The Centers for Disease Control and Prevention (CDC) released a PPE burn rate calculator for hospitals to project future PPE supplies.^2^ This tool requires that hospital systems have an understanding of daily PPE usage patterns. This calculator also does not differentiate usage at various healthcare provider (HCP) and patient capacities. Vital factors that affect PPE supplies, namely decontamination, are not considered in the CDC PPE burn rate calculator. We designed a sampling approach to steward our current PPE supply, and we developed an institutional burn rate calculator to project usage patterns at various patient and/or provider volumes.

We implemented a sampling process that utilized providers already present on a dedicated COVID-19 patient intensive care unit (ICU). We sampled continuously from weekdays to weekends to capture multiple use patterns. We previously implemented patient safety officers (PSOs) on each unit caring for COVID-19 patients to monitor infection control practices (eg, donning and doffing of PPE under extended use and reuse policies). The PSOs completed a survey of all providers present on the unit on the day and night shifts. This survey collected the following information: provider role, number of patients under the HCP care, number of COVID-19/person under investigation (PUI) patients under HCP care, number of contacts with COVID-19/PUI patients, number of patients for which the HCP uses an N95 mask, number of N95 masks used during a shift, number of N95 masks sent for decontamination, number of N95 masks disposed during a shift, number of gowns used during a shift, number of gowns disposed during a shift, number of disposable face shields used during a shift, number of disposable face shields disposed during a shift, number of disposable eye protection measures worn during a shift, number of disposable eye protection measures disposed during a shift, and number of aerosol-generating procedures (AGP) for which the HCP participated for a COVID-19/PUI patient. Once sampling was completed, we were able to develop our calculator.

We first calculated the average number of HCPs by role per shift (day or night) by dividing the total number of providers by the number of shifts. Next, we determined the number of N95s used by role per shift by dividing the total N95s used by the total number of providers. Using the average number of providers by role (per shift) and the total N95s used by role (per shift), we estimated the N95 use rate by role per shift. We then aggregated the estimated N95 use rate by provider role per shift to determine the estimated total N95 use rate per shift. To calculate the burn rate, we determined the disinfection rate of N95 masks by HCP role per shift. The disinfection rate equals the total number of masks disinfected divided by the total N95s used by role per shift. This disinfection rate is then used as a constant to estimate the number of masks that are disinfected by HCP role per shift based on the estimated masks used by role per shift. The N95 burn rate is then calculated by subtracting the estimated number of N95s disinfected by HCP role per shift from the estimated N95 use rate by role per shift. From the sampled data, we also incorporated a functionality to determine the impact of proportionate changes in COVID-19/PUI patient volumes on N95 utilization.

Our model required several assumptions. We assumed that our data collection helped us establish a baseline for the number of HCPs (and roles) that would interact with our COVID-19/PUI patients and that this level of care (ie, HCP-to-patient ratio) would be maintained as our volumes fluctuate (such that HCP staffing is directly proportional to patient volume). For example, if 5 nurses are staffed per shift to treat 10 COVID-19/PUI patients, 50 nurses would be staffed per shift for 100 COVID/PUI patients and the N95 use and burn rates would proportionally increase as well. Lastly, our calculator does not capture changes in N95 disinfection and N95 use rates, which can be subject to change.

**Fig. 1.**
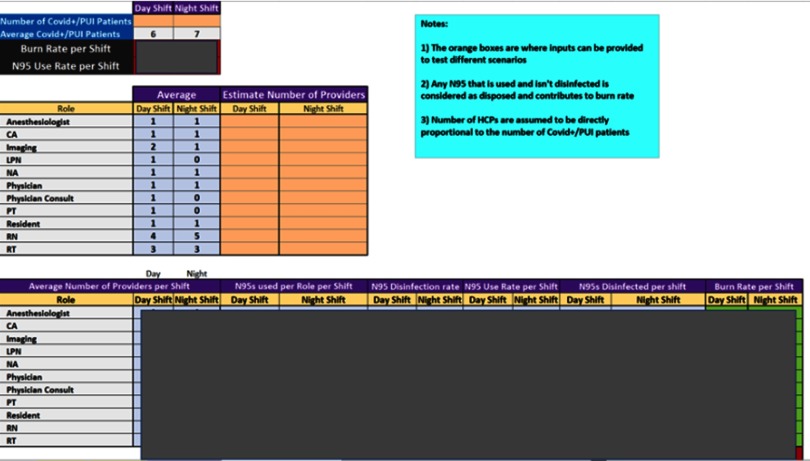
The personal protective equipment (PPE) burn rate calculator provides PPE usage rater based on healthcare provider role.

Our sampling process captured 158 providers over a total of 84 hours and 14 shifts: 6 day shifts (7:00 am through 7:00 pm) and 8 night shifts (7:00 pm through 7:00 am). We were able to track all providers that cared for COVID-19/ PUI patients on a single unit and thus to approximate N95 use on other dedicated COVID-19/PUI units. We also determined our N95 use rate, which informed future planning to optimize our inventory. Based on sampled data, the calculator can estimate N95 burn rates for simulated provider and patient volumes (Fig. 1).

We have demonstrated a novel sampling methodology that can be easily implemented to aggregate PPE use data. We also developed a Microsoft Excel tool that can allow administrators to reasonably prognosticate future supply and demand for PPE. Though we only sampled one unit, this tool also can allow users to sample more units and generate tighter confidence intervals on burn and use rates. At our institution, PSOs were already stationed on units performing other tasks, which aided data collection.

By examining provider use patterns, we can implement education initiatives that target specific provider roles. Our sampling method will benefit from validation, and we intend to continue to feedback our findings in other units to refine the output. We hope that our findings can assist other hospitals in easily determining their PPE burn rates and prolong their current supplies of PPE.
